# 95233 Analysis of 5'UTR Variation in Rare Disease Patients Reveals Variants of Potential Disease Relevance

**DOI:** 10.1017/cts.2021.659

**Published:** 2021-03-30

**Authors:** Bradley Bowles, Karl Clark, Eric Klee

**Affiliations:** 1 Department of Clinical and Translational Science, Mayo Clinic, Rochester, MN, USA; 2 Department of Biochemistry and Molecular Biology, Mayo Clinic, Rochester, MN, USA; 3 Biomedical Statistics & Informatics, Department of Health Sciences Research, Mayo Clinic, Rochester, MN, USA

## Abstract

ABSTRACT IMPACT: This work sheds diagnostic insight on patients with idiopathic rare disease and has the potential to further their care and treatment as a result. OBJECTIVES/GOALS: Correct diagnosis is imperative to treating patients with idiopathic, suspected genetic conditions, yet sequencing approaches leave up to 70% of these patients undiagnosed. We sought to improve diagnosis rates for a cohort patients referred for sequencing by characterizing deleterious variants within the 5’UTR. METHODS/STUDY POPULATION: We retrospectively analyzed whole exome sequencing (WES) data from 472 unsolved rare disease patients within the Mayo Clinic Center for Individualized Medicine to identify variants within the 5’UTR that affect the presence of upstream open reading frames (uORFs). uORFs are short regions (typically 30bp - 600bp) that typically influence downstream gene translation by sequestering ribosomes. We specifically searched for variants with the potential to disrupt existing uORFs or introduce new uORFs within the 5’UTR, and developed a pipeline to annotate these variants with information including GnomAD allele frequency and gene loss of function intolerance (pLI) score. To aid in variant interpretation, we applied two deep learning tools to predict variant impacts on transcript ribosome load (TITER and FramePool). RESULTS/ANTICIPATED RESULTS: Our pipeline identified a median of 21 variants per patient that were predicted to have a deleterious impact on the translational efficiency of protein coding transcripts, primarily by introducing new start codons within the 5’UTR or by altering the Kozak consensus of existing start codons. A median of 10 of these variants occur upstream of haploinsufficient genes with an existing disease association. We also identified a subset of variants that are predicted to introduce translationally active N-terminal extensions to protein coding transcripts, with the potential to disrupt protein localization and processing. DISCUSSION/SIGNIFICANCE OF FINDINGS: This work demonstrates that analysis of 5’UTR variants can be incorporated into existing WES pipelines, and identifies a group of variants with potential significance to patient disease. Further experimental evidence is necessary to ascertain the pathogenicity of these variants.

